# Metabolomic Profiles in Adipocytes Differentiated from Adipose-Derived Stem Cells Following Exercise Training or High-Fat Diet

**DOI:** 10.3390/ijms22020966

**Published:** 2021-01-19

**Authors:** Seita Osawa, Hisashi Kato, Yuki Maeda, Hisashi Takakura, Junetsu Ogasawara, Tetsuya Izawa

**Affiliations:** 1 Graduate School of Health and Sports Science, Doshisha University, 1–3 Tatara-Miyakodani, Kyoto 610–0394, Japan; seitapan@gmail.com (S.O.); hkato@mail.doshisha.ac.jp (H.K.); ctvd0003@mail4.doshisha.ac.jp (Y.M.); htakakur@mail.doshisha.ac.jp (H.T.); 2 Organisation for Research Initiatives and Development, Doshisha University, 1–3 Tatara-Miyakodani, Kyoto 610–0394, Japan; 3 Division of Health Science, Asahikawa Medical University, 2–1-1–1 Midorigaoka-Higashi, Hokkaido 078–8510, Japan; junetsu@asahikawa-med.ac.jp

**Keywords:** adipose-derived stem cell, exercise training, amino acids, perigonadal adipose tissue, subcutaneous adipose tissue, metabolome

## Abstract

Controlling the differentiation potential of adipose-derived stem cells (ADSCs) is attracting attention as a new strategy for the prevention and treatment of obesity. Here, we aimed to observe the effect of exercise training (TR) and high-fat diet (HFD) on the metabolic profiles of ADSCs-derived adipocytes. The rats were divided into four groups: normal diet (ND)-fed control (ND-SED), ND-fed TR (ND-TR), HFD-fed control (HFD-SED), and HFD-fed TR (HFD-TR). After 9 weeks of intervention, ADSCs of epididymal and inguinal adipose tissues were differentiated into adipocytes. In the metabolome analysis of adipocytes after isoproterenol stimulation, 116 metabolites were detected. The principal component analysis demonstrated that ADSCs-derived adipocytes segregated into four clusters in each fat pad. Amino acid accumulation was greater in epididymal ADSCs-derived adipocytes of ND-TR and HFD-TR, but lower in inguinal ADSCs-derived adipocytes of ND-TR, than in the respective controls. HFD accumulated several metabolites including amino acids in inguinal ADSCs-derived adipocytes and more other metabolites in epididymal ones. Kyoto Encyclopedia of Genes and Genomes enrichment analysis revealed that TR mainly affected the pathways related to amino acid metabolism, except in inguinal ADSCs-derived adipocytes of HFD-TR rats. These findings provide a new way to understand the mechanisms underlying possible changes in the differentiation of ADSCs due to TR or HFD.

## 1. Introduction

Adipose-derived stem cells (ADSCs), which are mesenchymal stem cells, are capable of differentiating into numerous phenotypes of mature cells, such as adipocytes, chondrocytes, myoblasts, osteoblasts, and other connective cells [1,2]. It has been considered that quality and cellularity of adipocytes in adipose tissue are regulated through the cycle of cell death and ADSC-induced cell neogenesis [3]. Indeed, reportedly, overweight status causes functional abnormalities in the differentiation capability of ADSCs to mature adipocytes, thereby disrupting the anaplerosis reaction of adipocytes, which have a normal cellular function, in the adipose tissue [4,5]. 

Visceral white adipose tissue (VAT) and subcutaneous white adipose tissue (SAT) have different characteristics and functional roles in metabolic regulation [6,7,8]. Excessive accumulation of VAT induces insulin resistance, with increased inflammatory response and oxidative stress, whereas accumulation of SAT has beneficial effects on the metabolism, as evidenced by the finding that intra-abdominal transplantation of SAT ameliorates body weight gain and insulin resistance [8,9,10]. These features of VAT and SAT are likely due to intrinsic and distinct functions of ADSCs in each fat pad in terms of adipogenesis and proinflammatory functions [8,11,12,13,14,15,16], and therefore, dysregulation in the functions and differentiation of ADSCs in each adipose tissue may lead to the development of obesity-based metabolic syndrome. Thus, elucidation of the mechanism underlying the differentiation of ADSCs in each fat pad as well as establishment of strategies for preventing obesity is needed. Notably, elucidation of the mechanisms underlying the differentiation of ADSCs in each fat pad via exercise training (TR) should be useful in establishing a new exercise therapy for the prevention and treatment of obesity. Recent advances indicate that preconditioning with TR induces higher ADSC retention [17], higher proangiogenic milieu of ADSCs [18], and mitigation effects against high-fat diet (HFD)-induced impairment of neurogenesis in ADSCs [19]. Furthermore, TR has previously been described to blunt the differential ability of epididymal ADSCs into adipocytes [20] and enhance the expression levels of vascular endothelial growth factor receptor 2 and vascular endothelial growth factor-A mRNAs in stromal vascular fraction (SVF) cells [21]. These results imply that TR has an impact on the differentiation potential of ADSCs in each fat pad and might play a different role in the pathogenesis of obesity. 

Growing evidence suggests that the pluripotency and differentiation of stem cells are mediated by their intracellular metabolic program [22,23,24,25]. Both naïve and primed pluripotent stem cells significantly depend on the glycolytic metabolism unlike somatic cells, and during differentiation, the metabolic flux of glycolysis and the pentose phosphate pathway is reduced, while oxidative phosphorylation in mitochondria is advanced [26,27,28]. Adipogenic induction of human ADSCs also requires more oxygen and increases mitochondrial activity, indicating organelle maturation and a transition from glycolytic to oxidative energy metabolism [29]. Furthermore, the differentiation of either preadipocytes [30] or ADSCs [31] into adipocytes is also accompanied by catabolism of branched-chain amino acids (BCAA) or accumulation of amino acids, respectively, as in the case of other stem cells. Amino acid metabolism plays a critical role in the energy metabolism of both embryonic stem (ES) and induced pluripotent stem cells [32,33,34] and has an impact on the function and differentiation of ES cells [29]. Given that metabolism in ADSCs is also central to their differentiation, it is hypothesized that as TR or HFD affects the differentiation potential of ADSCs [17,18,19,20], the metabolic profile of adipocytes differentiated from ADSCs may also change according to each intervention, and that these changes could possibly be different between adipocytes differentiated from ADSCs derived from VAT and SAT. However, a comprehensive metabolome analysis of adipocytes differentiated from ADSC of each fat pad after TR and/or HFD regimen has not been reported so far. 

The present study was designed to verify the above-mentioned hypothesis by metabolomic analysis of adipocytes differentiated from ADSCs of inguinal (inguinal ADSC-derived adipocytes) and epididymal adipose tissue (epididymal ADSCs-derived adipocytes), under stimulation with catecholamine, in the rats receiving TR or HFD, or both interventions. The analyses were performed for normal diet (ND)-fed control (ND-SED), HFD-fed control (HFD-SED), ND-fed TR (ND-TR), and HFD-fed TR (HFD-TR) groups. The metabolomic analysis was performed under stimulation with catecholamine because activation of β-adrenergic receptors is a candidate for the prevention of obesity and because exercise is well known to stimulate β-adrenergic receptors via rigorous activation of the sympathetic nervous system [35,36].

## 2. Results

### 2.1. Physical Characteristics of Animals

The final body weight (g, mean ± SE, *n* = 3 in each) was significantly lower in ND-TR (294.7 ± 7.6) and HFD-TR (303.0 ± 8.7) rats than in ND-SED (334.0 ± 11.8) and HFD-SED (366.3 ± 7.6) rats, respectively, at the 9-week time point of TR. The final masses (g, mean ± SE, *n* = 3 in each) of epididymal and inguinal adipose tissues in ND-TR (4.97 ± 0.36 and 4.53 ± 0.37, respectively) were also significantly lower than those in ND-SED (6.75 ± 0.97 and 7.77 ± 1.30, respectively). Each was also lower in HFD-TR rats (epididymal, 7.51 ± 0.48; inguinal, 6.71 ± 0.98) than in HFD-SED (epididymal, 13.00 ± 0.67; inguinal, 11.00 ± 0.48) rats.

### 2.2. Cellular Phenotypes of Adipocytes Differentiated from ADSCs

**Figure 1:**
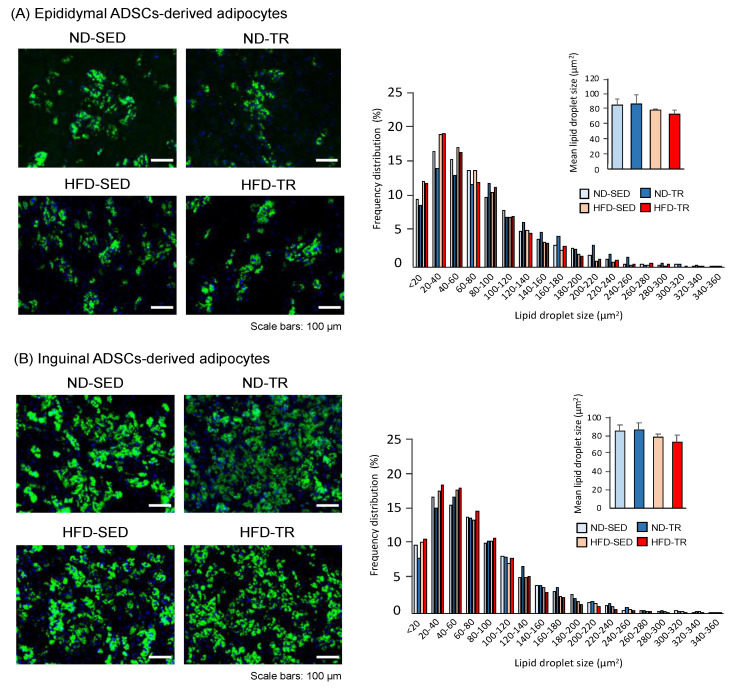
The images of typical cells differentiated from adipose-derived stem cells (ADSCs) and the size of lipid droplets. (**A**) Fluorescence microscope images of adipocytes differentiated from epididymal or (**B**) inguinal ADSCs, with DAPI and BODIPY staining showing nuclei and lipid droplets, respectively. Scale bars: 100 μm. Two right panels represent changes in the size of lipid droplet and frequency distribution of lipid droplet size in adipocytes differentiated from each respective ADSCs. A total of 10 different areas per experiment were analyzed for the size of lipid droplets. Data are presented as means ± standard deviations for mean lipid droplet size (*n* = 3 in each group), and as means for frequency distribution (*n* = 3). Lipid droplet sizes were clustered in ascending 25-μm^2^ intervals. ND, normal diet-fed; HFD, high-fat diet; C, sedentary rat; T, exercise-trained rat.

The prepared ADSCs were positive for the recommended markers (CD73^+^CD90^+^CD105^+^) [37], and no negative marker (CD45^−^) [37] was observed (Supplementary Figure S1). The images of typical adipocytes showing the size of cells are presented in Figure 1. Although we did not measure parameters related to the rate of differentiation of isolated ADSCs into adipocytes and their functions, for example, morphology, population doubling time, and separation time during passage, the formation of clear lipid droplets in adipocytes differentiated from ADSCs of each pad was evident (Figure 1). Both the mean and the distribution of BODIPY-stained lipid droplet size were not significantly different among the groups in epididymal ADSCs- (Figure 1A) and inguinal ADSCs-derived adipocytes (Figure 1B).

### 2.3. Metabolomic Analysis

**Figure 2:**
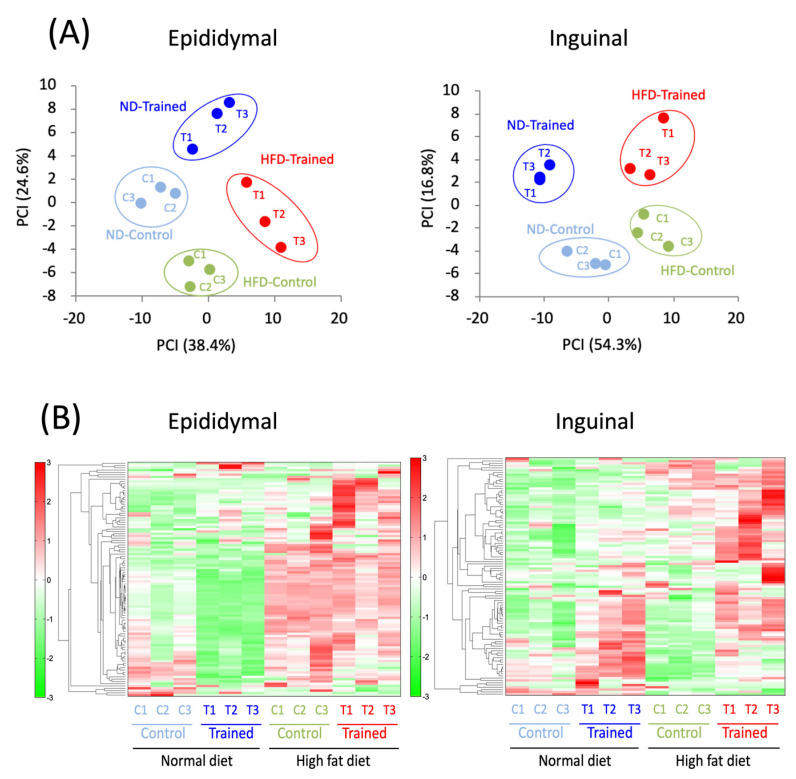
Overall qualitative and quantitative analysis of the metabolomics data. (**A**) Principal component analysis (PCA) of the metabolomic datasets of adipocytes differentiated from epididymal and inguinal ADSCs among different groups. Three samples were used in each group. Plots of each group are clearly distinguished on the PC1 axis (*X*-axis). (**B**) A heat map comparing metabolites. The color red indicates that the relative contents of metabolites are high, whereas the color green indicates that the relative contents of metabolites are low. ND, normal diet-fed; HFD, high-fat diet; C, sedentary rat; T, exercise-trained rat.

For the reasons mentioned in Section 1, we examined possible changes induced by isoproterenol (ISO) in metabolite profiles of inguinal ADSCs- and epididymal ADSCs-derived adipocytes. The heat map analysis (Figure 2A) and the principal component analysis (PCA) (Figure 2B) revealed that adipocytes differentiated from ADSCs of both fat pads in rats from each group segregated into four clusters. The effects of TR or HFD were engraved in ADSCs before adipocyte differentiation, resulting in in vitro differentiated adipocytes expressing metabolic properties unique to each intervention.

### 2.4. Effect of TR on the Accumulation of Metabolites in Adipocytes Differentiated from ADSC in ND Rats

**Figure 3:**
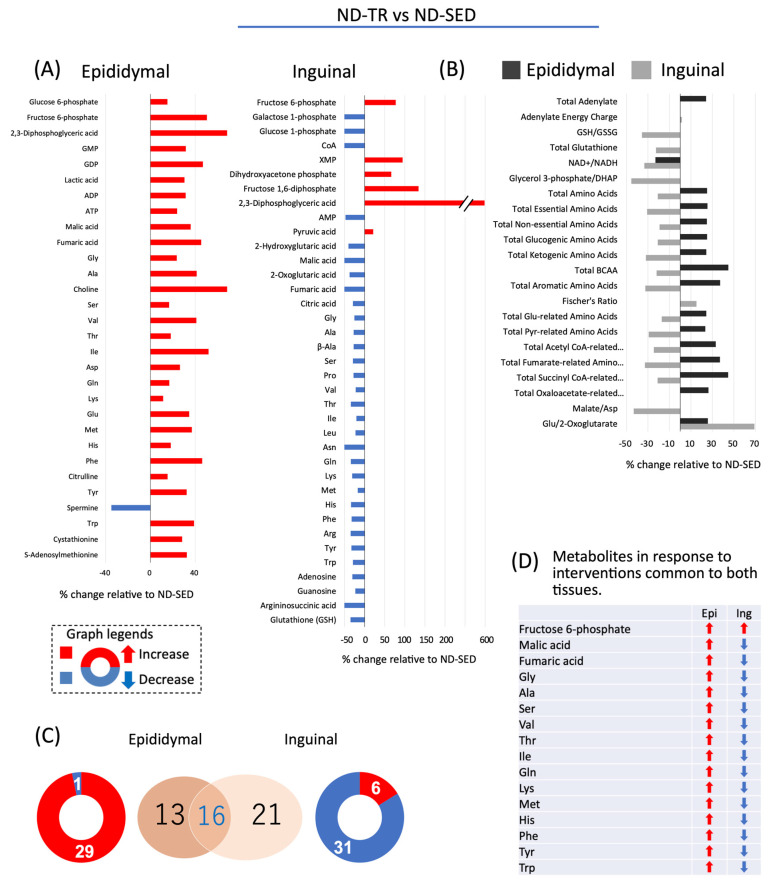
The response of metabolites to TR under ND intake in adipocytes differentiated from ADSC of each fat depot. (**A**,**B**) Changes in metabolites relative to ND-SED at a *p*-value cut-off of 0.05. (**C**) Venn diagram showing ND-TR vs. ND-SED. (**D**) Changes in the overlapped metabolites in adipocytes differentiated from ADSCs of each fat depot.

**Figure 4:**
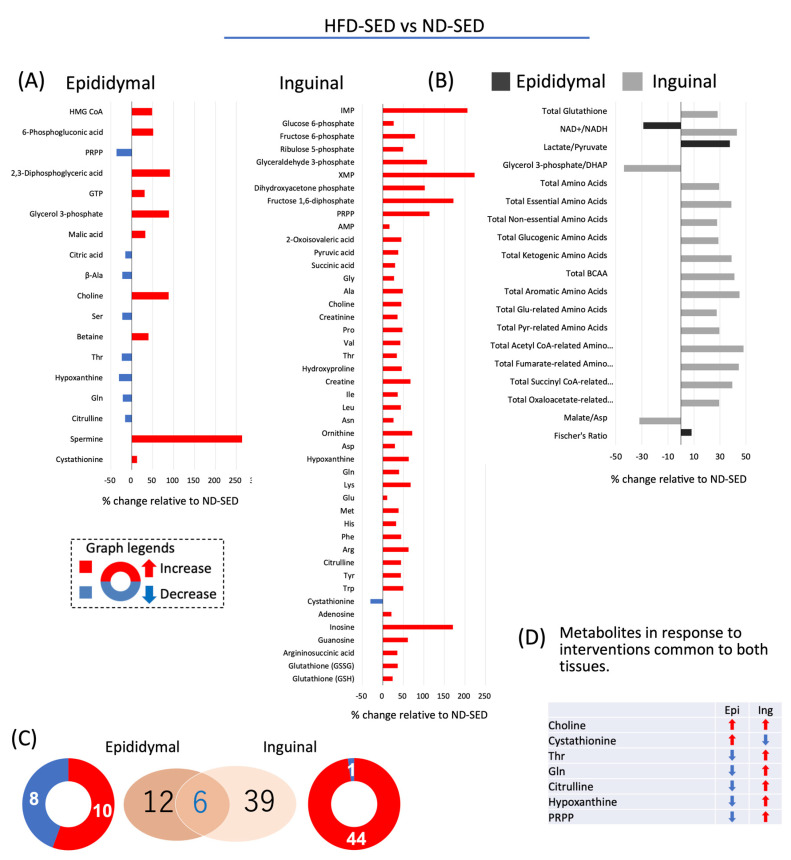
The response of metabolites to HFD in adipocytes differentiated from ADSCs of each fat depot. (**A**,**B**) Changes in metabolites relative to ND-SED at a *p*-value cut-off of 0.05. (**C**) Venn diagram showing HFD-SED vs. ND-SED. (**D**) Changes in the overlapped metabolites in adipocytes differentiated from ADSCs of each fat depot.

**Figure 5:**
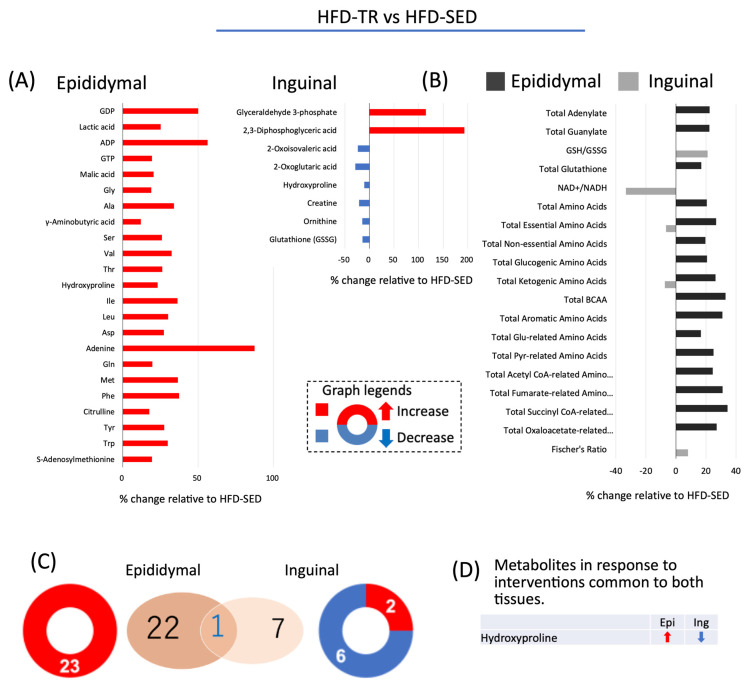
The response of metabolites to TR under HFD intake in adipocytes differentiated from ADSCs of each fat depot. (**A**,**B**) Changes in the levels of metabolites in response to HFD at a *p*-value cut-off of 0.05. (**C**) Venn diagram showing HFD-SED vs. HFD-TR. (**D**) Changes in the overlapped metabolites in adipocytes differentiated from ADSCs of each fat depot.

In the metabolomic analysis, 116 metabolites were analyzed (Tables S1 and S2: List and relative ratio of metabolites). Of the detected and calculated metabolic products, the metabolites that exhibited significant changes relative to each control rat are shown in Figure 3, Figure 4 and Figure 5. As shown in Figure 3A, in epididymal ADSCs-derived adipocytes of ND-TR rats, levels of 29 metabolites were increased and that of one metabolite was decreased relative to the levels in ND-SED rats. In contrast, in inguinal ADSCs-derived adipocytes of ND-TR rats, levels of six metabolites were increased and those of 31 metabolites were decreased relative to the respective levels in ND-SED rats. As shown in Figure 3B, TR under ND intake increased cellular accumulation of amino acids associated with energy production in epididymal ADSCs-derived adipocytes but reduced the accumulation in inguinal ADSCs-derived adipocytes. A decrease in the NAD^+^/NADH ratio was observed in both the type of differentiated adipocytes. Sixteen metabolites, levels of which changed, were common in epididymal ADSCs-derived and inguinal ADSCs-derived adipocytes, but cellular accumulation of these metabolites, except for fructose 6-phosphate and 2,3-diphosphoglyceric acid, exhibited an opposite pattern (Figure 3C,D).

### 2.5. Effect of HFD on Cellular Accumulation Levels of Metabolites in Adipocytes Differentiated from ADSCs

As shown in Figure 4A, in epididymal ADSC-derived adipocytes of HFD-SED rats, levels of 10 metabolites were increased and those of eight metabolites were decreased relative to the respective levels in those derived from ND-SED rats. In inguinal ADSCs-derived adipocytes of HFD-SED rats, levels of 44 metabolites were increased, and that of one metabolite was decreased relative to the respective levels in those derived from ND-SED rats. As shown in Figure 4B, HFD increased the cellular amino acid contents compared with ND-SED rats in inguinal ADSCs-derived adipocytes but not in epididymal ADSCs-derived adipocytes: HFD increased cellular accumulation of amino acids associated with energy production in inguinal ADSCs-derived adipocytes (Figure 4B).

Epididymal ADSCs-derived adipocytes of HFD-SED rats, compared with those from ND-SED rats, exhibited increased lactate/pyruvate ratio with a decreased NAD^+^/NADH ratio, whereas inguinal ADSCs-derived adipocytes showed an increase in NAD^+^/NADH ratio without a significant change in the lactate/pyruvate ratio (Figure 4B). Thus, the effect of HFD on these two parameters was opposite for inguinal ADSCs-derived and epididymal ADSCs-derived adipocytes. Six metabolites, the levels of which changed, were common in epididymal ADSCs- and inguinal ADSCs-derived adipocytes; compared with the metabolites of ND-SED rats, the levels of Thr, Gln, citrulline, hypoxanthine, and 5-phosphoribosyl 1-diphosphate (PRPP) increased and decreased in inguinal ADSC- and epididymal ADSCs-derived adipocytes of HFD-SED rats, respectively; the levels of cystathionine decreased and increased in inguinal ADSCs- and epididymal ADSCs-derived adipocytes of HFD-SED rats, respectively; the levels of choline increased in both inguinal ADSCs- and epididymal ADSCs-derived adipocytes of HFD-SED rats (Figure 4C,D).

### 2.6. Effect of TR on Cellular Accumulation Levels of Metabolites in Adipocytes Differentiated from ADSC in HFD Rats

In epididymal ADSCs-derived adipocytes of HFD-TR rats, levels of 23 metabolites were increased compared with those in HFD-SED rats (Figure 5A). In contrast, in inguinal ADSCs-derived adipocytes from HFD-TR rats, levels of two metabolites were increased, and those of six metabolites were decreased relative to those in inguinal ADSCs-derived adipocytes from HFD-SED rats. Thus, HFD-TR rats exhibited increased cellular amino acid contents compared with HFD-SED rats in epididymal ADSCs-derived adipocytes but not in inguinal ADSCs-derived adipocytes: TR increased cellular accumulation of amino acids associated with energy production in epididymal ADSCs-derived adipocytes. Furthermore, the NAD^+^/NADH ratio was decreased in inguinal ADSCs-derived adipocytes from HFD-SED rats compared with that in inguinal ADSCs-derived adipocytes from ND-SED rats (Figure 5B). TR altered the accumulation of two metabolites, ornithine and hydroxyproline, which were common in adipocytes differentiated from both epididymal ADSCs- and inguinal ADSCs-derived adipocytes, and the accumulation of two metabolites displayed opposite patterns between epididymal ADSCs- and inguinal ADSCs-derived adipocytes (Figure 5C,D). 

### 2.7. Kyoto Encyclopedia of Genes and Genomes (KEGG) Enrichment Analysis 

**Figure 6:**
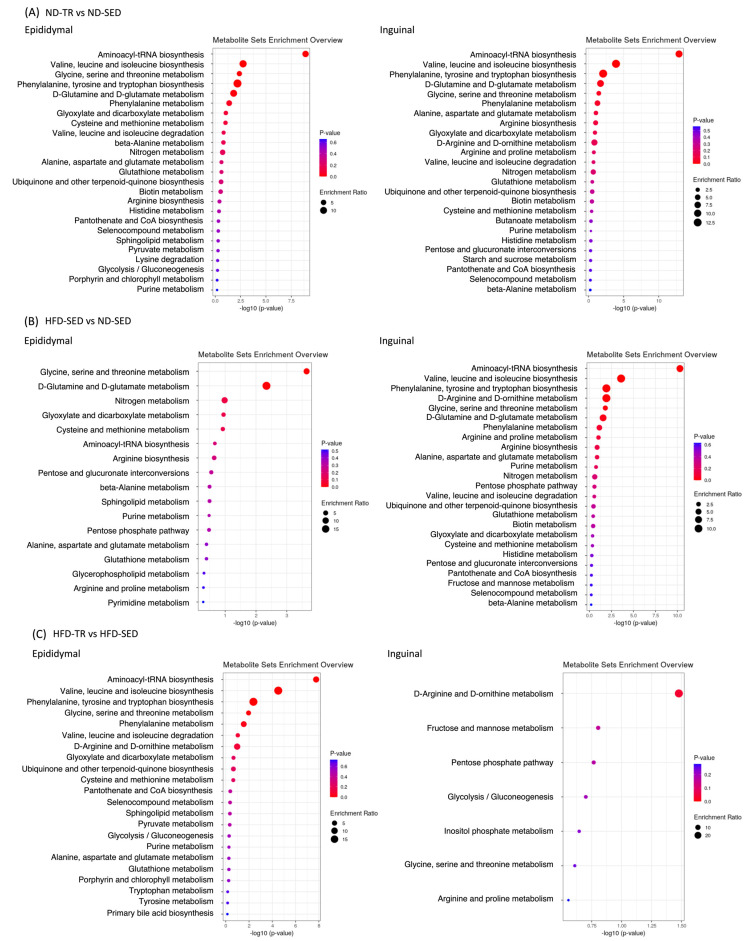
The pathway impact in topology analysis and *p*-value in enrichment analysis conducted via Metabolomics Pathway Analysis (MetPA, www.metaboanalyst.ca) based mainly on the KEGG metabolic pathways. (**A**) ND-TR vs. ND-SED. (**B**) HFD-TR vs. HFD-SED. (**C**) ND-SED vs. ND-TR. The size of pathway symbols represents the significance level of enrichment analysis, and the color of pathway symbols represents the impact factor. ND-SED, normal diet-fed sedentary controls; HFD-SED, high-fat diet-fed sedentary controls; ND-TR, normal diet-fed exercise-trained; HFD-TR, high-fat diet-fed exercise-trained group.

We next examined the differences and dynamic changes in biological processes among ND-TR vs. ND-SED, HFD-SED vs. ND-SED, and HFD-TR vs. HFD-SED comparisons using metabolomics pathway analysis based mainly on KEGG (Figure 6). As expected, TR mainly affected the pathways related to amino acid metabolism except for inguinal ADSCs-derived adipocytes from HFD-TR rats. The top three pathways in SED vs. TR comparison were as follows; in epididymal ADSCs-derived adipocytes from ND-TR vs. ND-SED (Figure 6A), aminoacyl-tRNA biosynthesis, valine/leucine/isoleucine biosynthesis, glycine/serine/threonine metabolism; in inguinal ADSCs-derived adipocytes from ND-TR vs. ND-SED (Figure 6A), aminoacyl-tRNA biosynthesis, valine/leucine/isoleucine biosynthesis, phenylalanine/tyrosine/tryptophan biosynthesis; in epididymal ADSCs-derived adipocytes from HFD-TR vs. HFD-SED (Figure 6C), aminoacyl-tRNA biosynthesis, valine/leucine/isoleucine biosynthesis, phenylalanine/tyrosine/tryptophan biosynthesis; in inguinal ADSCs-derived adipocytes from HFD-TR vs. HFD-SED (Figure 6C), D-arginine/D-ornithine metabolism, fructose/mannose metabolism, pentose phosphate pathway. In HFD-SED vs. ND-SED comparison (Figure 6B), HFD affected glycine/serine/threonine metabolism, D-glutamine/D-glutamate metabolism, and nitrogen metabolism in epididymal ADSCs-derived adipocytes from HFD-SED rats, whereas it affected aminoacyl-tRNA biosynthesis, valine/leucine/isoleucine biosynthesis, and phenylalanine/tyrosine/tryptophan biosynthesis were mainly in inguinal ADSCs-derived adipocytes from HFD-SED rats. 

Taken altogether, the effects of HFD and TR on ADSCs were prominent in the intracellular amino acid metabolism of in vitro differentiated adipocytes, and these effects on the cellular accumulation of amino acids displayed opposite patterns between epididymal ADSCs- and inguinal ADSCs-derived adipocytes. Compared with the respective ND-SED rats, higher accumulations of amino acids were found along with enhanced biosynthesis of amino acids determined using KEGG metabolic analysis in epididymal ADSCs-derived adipocytes of ND-TR rats, whereas inguinal ADSCs-derived adipocytes of ND-TR rats displayed contrasting results.

### 2.8. Metabolism of the Tricarboxylic Acid (TCA) Cycle-Related Amino Acids 

**Figure 7:**
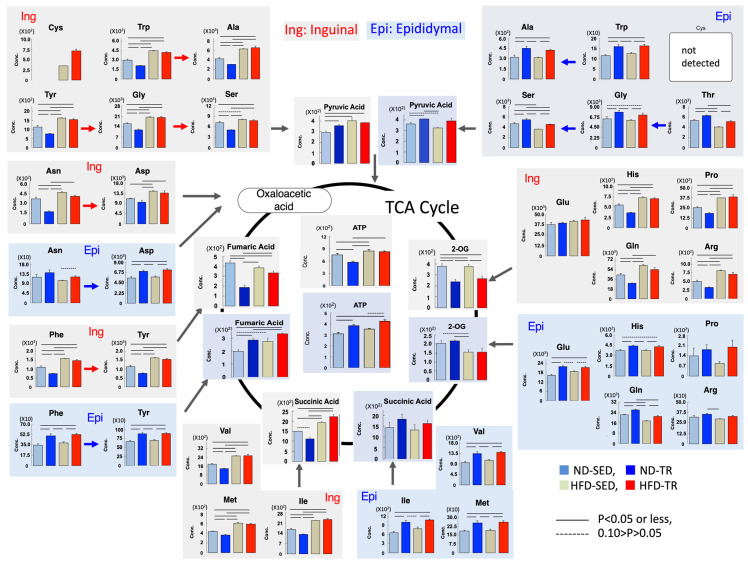
The concentrations (pmol/10^6^ cells) of ATP, amino acids, and the metabolites of the TCA cycle in adipocytes differentiated from inguinal (Ing) and epididymal (Epi) ADSCs (upper panel). Data are expressed as mean ± SD (*N* = 3); solid lines and dotted lines indicate *p* < 0.05 or less, and 0.10 > *p* > 0.05, respectively. The lower table shows the ratio of metabolites. 2-OG, 2-oxoglutaric acid; ND-SED, normal diet-fed sedentary controls; HFD-SED, high-fat diet-fed sedentary controls; ND-TR, normal diet-fed exercise-trained; HFD-TR, high-fat diet-fed exercise-trained group.

Based on the findings mentioned, we determined the possible relationships between the levels of amino acids and those of metabolites related to the TCA cycle (Figure 7, Table 1). Under ND intake, in epididymal ADSCs-derived adipocytes of the ND-TR group, significant elevation of fumaric acid and ATP was observed, and amino acids were elevated compared with that in the ND-SED group. In contrast, inguinal ADSCs-derived adipocytes in ND-TR group, compared with those in the ND-SED group, exhibited lower accumulations of 2-oxoglutaric acid, succinic acid, fumaric acid, and ATP, with lower accumulations of amino acids except for Glu. Under HFD intake, there were no significant differences in the levels of amino acids in inguinal ADSCs-derived adipocytes between the HFD-TR and HFD-SED groups, but in epididymal ADSCs-derived adipocytes, accumulations of amino acids, except for Pro and Arg, were increased. However, no significant change was found in the accumulation of ATP and metabolites of the TCA cycle between the HFD-SED and HFD-TR groups. Thus, TR-induced changes in the levels of amino acids were the same as inguinal ADSCs- and epididymal ADSCs-derived adipocytes.

As shown in Table 1, the ratio of metabolites indicates the differences in metabolic profiles between inguinal ADSCs- and epididymal ADSCs-derived adipocytes, and the differences in the effects of exercise on these differences. Epididymal ADSC-derived adipocytes from ND-TR rats had a lower free NAD^+^/NADH ratio and a higher Glu/2-oxoglutarate ratio in response to ISO compared with those from ND-SED rats, suggesting enhanced glycolysis and hypoxia in the presence of ISO. In response to ISO, inguinal ADSCs-derived adipocytes also exhibited a possible increase in glycolysis and hypoxia as evidenced by a lower free NAD^+^/NADH ratio and a higher Glu/2-oxoglutarate ratio compared with those from ND-SED rats, but unlike epididymal ADSCs-derived adipocytes, inguinal ADSCs-derived adipocytes had a lower malate/Asp ratio in response to ISO, implying an inhibition of amino acid catabolism into metabolites associated with the TCA cycle.

HFD induced a lower NAD^+^/NADH ratio and a higher lactate/pyruvate ratio in epididymal ADSCs-derived adipocytes in response to ISO. The result suggests enhanced glycolysis in response to ISO. In contrast, inguinal ADSCs-derived adipocytes of HFD-SED rats exhibited a higher NAD^+^/NADH ratio, a lower GSH/GSSH ratio, and a lower malate/Asp ratio compared with the respective ratios in ND-SED rats under ISO stimulation. Thus, HFD may induce a reduction in glycolysis and an inhibition of amino acid catabolism in TCA cycle with enhanced oxidative stress in the presence of ISO. As expected, TR induced an improvement in oxidative stress and an increase in glycolysis in inguinal ADSCs-derived adipocytes in response to ISO, as evidenced by a lower NAD^+^/NADH ratio and a higher GSH/GSSH ratio. However, no significant change in the parameters was found in epididymal ADSCs-derived adipocytes between HFD-SED and HFD-TR rats in response to ISO.

## 3. Discussion

Adipocytes differentiated from ADSCs of epididymal and inguinal adipose tissues after TR and/or HFD interventions displayed different metabolic profiles from each sedentary control under isoproterenol stimulation. In particular, TR affected the cellular accumulation of amino acids in a fat depot-specific manner. TR increased the cellular accumulations of amino acids in the presence of ISO in epididymal ADSCs-derived adipocytes regardless of the diet type in rats, whereas it decreased the accumulation in inguinal ADSCs-derived adipocytes from ND-fed rats but not from HFD-fed rats. To the best of our knowledge, these comparative results of metabolic profiles are reported for the first time.

Amino acids are metabolized and integrated into the TCA cycle [38]. Essential and nonessential amino acids are degraded to products that can be metabolized for energy production. TR-induced high or low accumulation of amino acids showed the same behavior as the changes in the levels of TCA cycle metabolites in epididymal ADSCs-derived adipocytes, regardless of the diet. These results imply that the degradation and synthesis of amino acids in the presence of ISO could be enhanced in epididymal ADSCs-derived adipocytes from ND-TR and HFD-TR rats. Indeed, epididymal ADSCs-derived adipocytes from ND-TR rats had a lower free NAD^+^/NADH ratio with greater accumulation of ATP and lactate compared with those from ND-SED rats. This notion could be supported in part by the presence of hypoxia as implied by a higher Glu/2-oxoglutaric acid ratio compared to that in ND-SED rats and the reported upregulation of hypoxia-inducible factor-1α in epididymal SVF-derived adipocytes [20]. The expression levels of *Glut4*, *Pgam2*, and *Pkm* mRNAs for glucose uptake and glycolysis are enhanced in the perigonadal SVF-derived adipocytes of exercise-trained mice [39]. This robust study alternatively suggests an enhanced function of mitochondria in the perigonadal SVF-derived adipocytes of exercise-trained mice, as well as the increased expression of several mitochondrial and ‘beiging’ genes (*Cpt1*, *Nrf1*, *Nrf2*, and *Prdm16*) and the increased expression of genes involved in fatty acid oxidation and uptake (*Fabp3*, *Acsl1*, *Acsl3*, *Acsl4*, *Acsl5*, *Gyk*) [39]. Consequently, our data imply that in epididymal ADSCs-derived adipocytes of ND-TR rats, glycolysis was used in preference to the TCA cycle for energy production in the presence of ISO. However, HFD diet may also enhance glycolysis in epididymal ADSCs-derived adipocytes in response to ISO, but epididymal ADSCs-derived adipocytes from HFD-TR rats had a tendency of greater accumulation of ATP (*p* = 0.06) without a significant change in these parameters compared with HFD-SED rats, suggesting that glycolysis and the TCA cycle activity might be more balanced in HFD-TR rats than those in HFD-SED rats. 

Inguinal ADSCs-derived adipocytes from ND-TR rats, in contrast, had an inhibition of catabolism of amino acids into metabolites associated with the TCA cycle compared with those of ND-SED rats. These results were incongruous with those reported previously. The reported potential of ADSCs for beige adipocyte differentiation [40,41] should be associated with the rigorous activity of mitochondria; adipocytes differentiated from inguinal SVF-derived cells of exercise-trained mice displayed higher basal oxygen consumption rate, ATP turnover, and maximal respiratory capacity, with increased expression of hexokinase 2, glucose transporter 1, glucose transporter 4, and basal glucose uptake, compared with those of sedentary mice. Furthermore, inguinal ADSCs-derived adipocytes from HFD-TR rats were less sensitive to TR than epididymal ADSCs-derived adipocytes from HFD-TR rats. Further research is needed to elucidate these contradictions and fat depot-specific differences.

The above-mentioned differences between epididymal ADSCs- and inguinal ADSCs-derived adipocytes could also be influenced by higher adipocyte lipolysis in VAT compared with that in SAT in both trained [42] and sedentary lean rats [42,43]. During stimulation of lipolysis by ISO, a large population of the released free fatty acids could be reincorporated into adipocytes [44,45,46], and catecholamine-induced suppression of re-esterification enhanced fatty acid oxidation [47,48,49]. However, the perigonadal SVF-derived adipocytes of exercise-trained mice had increased expression of genes involved in fatty acid oxidation and uptake [39]. In TR lean rats, the isolated epididymal adipocytes exhibited no significant difference relative to isolated inguinal adipocytes in the extent of palmitic acid oxidation [49] and activity of cytosolic enzymes involved in lipogenesis [50]. Therefore, based on the literature on lean rats, a possibility could be that stimulated lipolysis significantly modifies the differences in metabolites between epididymal ADSCs- and inguinal ADSCs-derived adipocytes after TR. However, to our knowledge, limited information is available regarding the effects of TR on lipogenesis and lipolysis in both visceral and subcutaneous adipocytes in the context of HFD-induced obesity. One study showed an inhibitory effect of TR on the lipolytic activity of adipocytes in obese rats, but this study also showed a blunted lipolysis in adipocytes in lean rats [51]. TR has been shown to improve mitochondrial functions with reduced lipogenic-related markers, sterol regulatory element-binding transcription factor 1c, and acetyl CoA carboxylase, in epididymal white adipose tissue, but this study did not cover subcutaneous adipose tissue [52]. To elucidate the mechanism behind the phenomenon observed by us, data for both the expression of enzymes related with metabolite changes and for the balance of synthesis and degradation of metabolites are required. Lack of these data do not allow us to comment on the regulation of metabolic pathways.

Amino acids play a major role in regulating the physiological functions of adipocytes, and thus fat depot-specific differences in the accumulation of amino acids may be involved in the TR-induced changes in several functions of adipocytes. First, these may account, in part, for our previous results that exercise training suppresses basal autophagy activity in epididymal white adipose tissue, but that this activity is enhanced in inguinal white adipose tissue [53]. Among several physiological signals activating mammalian target of rapamycin complex 1 (mTORC1), nutrients, particularly amino acids, are known to cause the activation of mTORC1, and mTORC1 signaling inhibits autophagy [54]. 

Furthermore, BCAAs are implicated in elevated peroxisome proliferator-activated receptor (PPAR) activity [55], whereas the expression levels of BCAA catabolic enzymes are controlled in part by PPAR and PPARγ coactivator 1-α (PGC-1α) [56,57]. Both proteins can enhance the expression and activity of BCAA-degradation enzymes in primary hepatocytes [57] or 3T3L1 adipocytes [58]. Furthermore, PPARγ, expression of which is down- and upregulated in epididymal SVF cells [20] and primary adipocytes [59], respectively, regulates BCAA-degradation enzymes in db/db mice [58]. PPARγ and PGC-1α play a crucial role in the induction of adipogenesis and mitochondrial biogenesis. In this context, it is of interest to consider that amino acid metabolism contributes to energy metabolism in both ES and induced pluripotent stem cells [30,31,32] and that BCAAs catabolism or accumulation of amino acids are strongly related to the differentiation of either preadipocytes [33] or ADSCs [34] into adipocytes, respectively. Obesity-derived human and murine ADSCs also reportedly indicate alterations in the carbohydrate metabolism and deep differences in lipid and amino acid catabolism, respectively [60]. This indicates that modulation of ADSCs metabolism might cause a possible change in the differentiation ability. Accordingly, it is tempting to speculate that the TR- or HFD-related change in the amino acid metabolism in adipocytes differentiated from ADSCs might be associated with the reported findings that TR can alter the differentiation potential of ADSCs into adipocytes [20,61] and neuron-like cells [19]. Furthermore, enrichment of aminoacyl-tRNA biosynthesis in both epididymal ADSCs- and inguinal ADSCs-derived adipocytes of ND-TR, and epididymal ADSCs-derived adipocytes of HFD rats might be reminiscent of changes in protein synthesis efficiency caused by TR in each type of diet. Aminoacyl-tRNAs are biologically active substrates for peptide bond formation in protein synthesis [62,63].

The different metabolic profiles in VAT and SAT are possible key determining factors for the response and accommodation to metabolic input for energy homeostasis including obesity-related metabolic diseases [6,7,8]. This is supported by the findings that reveal insulin resistance induced by excess accumulation of VAT and amelioration of body weight gain and insulin resistance upon intra-abdominal transplantation of SAT [8,9,10]. Such different metabolic profiles in each fat pad are ascribed to different intrinsic developmental origins with distinct adipogenic progenitors [8]. In this context, as discussed above, the present results indicate that one of the mechanisms underlying the reported effects of TR on each fat pad [20,39,41,53] likely involves the observed differences in metabolic profiles between epididymal ADSCs- and inguinal ADSCs-derived adipocytes. However, it is difficult to clearly propose that VAT and SAT play opposite roles in either the expansion of fat pads or the appearance of pathological conditions, for example, oxidative stress and inflammation, in metabolic syndrome [64,65,66]. In the present study, no significant difference in the lipid droplet size in differentiated adipocytes from ADSCs of each group indicates that the different accumulation of metabolites between groups did not always contribute to control the lipid droplet size under our experimental condition. Oxidative stress indicated by NAD^+^/NADH, NADP^+^/NADPH, and GSH/GSSH ratios also exhibited no change between epididymal ADSCs-derived adipocytes and subcutaneous adipocytes in other groups than in the HFD group. The beneficial effects of exercise on improving oxidative stress in adipocytes was only found in inguinal ADSCs-derived adipocytes as estimated by the GSH/GSHR ratio. These data are inconsistent with the TR-induced differences in expression of either the fatty acids metabolism-related molecules determining adipocyte size (for review, see [65]) or oxidative stress status [66,67], or both, between VAT and SAT. The discrepancies might be due to the assumption that adipocytes differentiated from ADSCs in vitro had been unaffected by factors that likely influence their differentiation in vivo within adipose tissue. For example, it is well known that oxidative stress and inflammatory response are the major causes of macrophage infiltration into the adipose tissue. 

The present study presents an interesting finding that adipocytes differentiated from ADSCs of either SAT or VAT of the rats preconditioned with TR or HFD present different trends of metabolite accumulation in response to ISO, but does not confirm that ADSCs could be differentiated into adipocytes in vivo in VAT and SAT on the basis of features observed by us. To establish the significance of the differences in metabolic accumulation in ADSCs and the possible candidates for the physiological roles controlling the effects of TR and HFD, further studies are required to comprehensively compare mature adipocytes and to analyze adipocytes that have differentiated under conditions where we can reproduce the niche environment in vivo in the adipose tissue. The present study has other significant technical limitations. First, metabolome analysis is helpful in detecting the accumulation of metabolites within cells, and thus, does not distinguish between synthesis and degradation. This weakness and lack of the data for the expression of enzymes related with the observed changes in metabolites do not allow an in-depth discussion on the regulation of metabolic pathways. Second, it should be noted that the sample size was relatively small for metabolome analysis. Third, the exact phenotype of ADSCs and differentiated adipocytes remains to be verified, although we confirmed the expression of specific markers in ADSCs and the presence of lipid droplets in adipocytes. Finally, it is well known that there are gender differences in the metabolism of adipose tissue [68]. The findings of the present study refer only to male rats, and thus it is necessary to perform investigations in female rats. Further studies are required to compensate for the above limitations.

## 4. Materials and Methods

### 4.1. Animal Care and TR Programme

Four-week-old male Wistar rats (*n* = 12; SLC, Shizuoka, Japan) were housed in a temperature-controlled room. All rats were given 7 days to acclimatize to their new environment. The rats were kept at a temperature of 23 °C with a 12 h:12 h light-dark cycle. The rats of ND groups were fed a standard normal diet containing 9% fat (MF; Oriental Yeast Co., Ltd., Tokyo, Japan) and the rats of the HFD groups were exposed to HFD containing 60% saturated fat (D12492; Research Diets, Inc., New Brunswick, NJ, USA). They were then randomly assigned to ND-SED (*n* = 3), HFD-SED (*n* = 3), ND-TR (*n* = 3), and HFD-TR (*n* = 3) groups for 9 weeks. Food and water were available ad libitum. The training rats were subjected to running on a treadmill set at a 5° incline 5 days per week for 9 weeks, as previously described [19,20,21,53,69,70,71]. The rats ran on a treadmill at 6:00, corresponding to the time point of the late part of the active phase according to our recent study [43]. The running speed and duration were progressively increased until after 6 weeks when the rats ran continuously at 30 m/min for 90 min. Both groups started with a warmup at 10 m/min, and thereafter, the pace and the time of continuous running were increased gradually from 15 to 30 m/min and 30 to 90 min over 9 weeks, respectively. The intensity of this training protocol was estimated to be 60–70% VO_2_ max [72,73,74] and enhance citrate-synthase activity [71] and adipocyte lipolysis in response to ISO [69,70]. The sedentary control rats were not subjected to treadmill running. 

At the end of the study, to avoid any influence of a last bout of exercise, the exercised rats were euthanized under anesthesia 36 h after the end of the last 90 min exercise session. Subsequently, the epididymal and inguinal adipose tissues were rapidly removed and enzymatically dispersed to obtain primary adipocytes from all groups. 

All animal protocols were approved by the Animal Care Committee of Doshisha University (A17010, A18058, A19005).

### 4.2. Isolation and Preparation of SVF Cells from Adipose Tissues

SVF cells containing ADSCs were isolated from epididymal and inguinal adipose tissue according to our previous study [8,9]. In brief, adipose tissues were minced with scissors and placed in plastic vials in the isolation buffer (Krebs–Ringer bicarbonate solution buffered with 10 mM HEPES, pH 7.4, containing 5.5 mM glucose and 2.5% (*w*/*v*) fatty acid-free bovine serum albumin) with 200 nM adenosine and collagenase type 1 (2 mg/mL; Wako Pure Chemical Industries, Ltd., Osaka, Japan). Collagenase digestion was performed at 37 °C in a water-bath shaker. After 45 min, the vials were filtered and centrifuged at 600× *g* for 10 min. SVF pellets were then washed twice with phosphate-buffered saline and seeded onto a 10-cm dish in Dulbecco’s modified Eagle medium (DMEM; Sigma-Aldrich, St. Louis, MO, USA) supplemented with 10% fetal bovine serum and 1% penicillin/streptomycin (Gibco, Grand Island, NY, USA), and maintained at 37 °C in a 5% CO_2_ atmosphere. At 80% confluence, the cultures were passaged using 0.25% Trypsin/EDTA (Gibco) and reseeded at a density of 1.0 × 10^4^ cells/cm^2^. SVF cells between passages three to four were used for subsequent experiments as ADSCs. Cultured SVF cells appeared as fibroblast-like cells and acquired a homogenous spindle shape after three passages, and their features were consistent with isolated ADSCs [75,76,77].

### 4.3. Reverse Transcriptase-Polymerase Chain Reaction (RT-PCR) Analysis 

The expression levels of ADSCs markers were examined by RT-PCR to verify the characteristics of the obtained ADSCs. First, total RNA was prepared using Isogen (Nippon Gene, Toyama, Japan) from a portion of the ADSCs. First-strand cDNA was obtained by incubating total RNA samples (2 μg) with reverse transcriptase (RT, Superscript III; Invitrogen, Carlsbad, CA, USA) in a reaction mixture (18 μL). The RT product (1 μL) was subjected to polymerase chain reaction (PCR) using Taq DNA polymerase (Applied Biosystems, Foster City, CA, USA). Thereafter, 20–35 cycles of amplification were carried out using the following conditions for each cycle: denaturing at 94 °C for 30 s, annealing at 55 °C for 60 s, and extension at 72 °C for 90 s. The PCR products were electrophoresed in 1% agarose gels containing ethidium bromide. When we decided appropriate cycles of PCR amplification to each gene, we confirmed that the cycles corresponded to the linear phase of PCR amplification by line graphs making with the intensity of bands from the RT-PCR. The sequences of primers used are mentioned as follows: CD45: 5′-AAAGACGAAATGGCTCCTCAG-3′, 5′-CTATTTCTGTGCTTGTGGTGG-3′; CD73: 5′-TGATAACGGTGTGGAAGGAC-3′, 5′-CTTCACGAATGGTGCTGTT-3′; CD90: 5′-CCTGCCTGGTGAACCAGAACCTT-3′, 5′-GCAGGCTTATGCCACCACACTGAC-3′; CD105: 5′-CAGGCATCCAACACCATAGAG-3′, 5′-AAGTTCATGGCCGATGGTTCC-3′. 

### 4.4. Differentiation of ADSC into Mature Adipocytes

In vitro differentiation of ADSC into mature adipocytes was performed using a protocol modified from the method of Sakurai et al. [9]. ADSC was seeded at a density of 1.0 × 10^5^ cells/cm^2^ into a 6-well plate in the growth medium, DMEM supplemented with 10% fetal bovine serum and 1% penicillin/streptomycin, and were allowed to grow to 100% confluence. After three washes with phosphate-buffered saline, the medium was changed to the growth medium supplemented with 500 µM 3-isobutyl-1-methylxanthine (Sigma-Aldrich), 1 μM dexamethasone (Sigma-Aldrich), 10 μg/mL insulin (Wako Pure Chemical Industries Ltd., Osaka, Japan), and 100 µM indomethacin (Wako). Three days later, the induction medium was replaced by the growth medium supplemented with 10 μg/mL insulin and 100 µM indomethacin until accumulation of the triglyceride content. The medium was renewed every three days. The sufficient lipid droplet formations were confirmed in inguinal ADSCs- and epididymal ADSCs-derived adipocytes with Adipocyte Fluorescent Staining kit (PMC, Hokkaido, Japan) at 9 and 12 days, respectively, as described in the next section. 

### 4.5. Fluorescent Staining and Size of Lipid Droplets

Adipogenic differentiation of ADSCs was examined for lipid accumulation by BODIPY (boron dipyrrin, Thermo Fisher Scientific, Tokyo, Japan) staining. Briefly, lipid droplets and nuclei were stained using the Adipocyte Fluorescent Staining kit (PMC, Hokkaido, Japan) according to the manufacturer’s protocol. In this experiment, a portion of cells was used before ISO stimulation. Briefly, the cells were washed once with washing buffer, and fixed overnight at room temperature with 10% formalin. Thereafter, formalin was removed, and cells were incubated for 30 min at room temperature with BODIPY. BODIPY was then washed off, and cells were stained for 30 min at room temperature with Hoechst 33258. Finally, cells were washed once with washing buffer, and treated with the mounting agent. Images were obtained with BZ-8100 fluorescent microscope (KEYENCE, Osaka, Japan). A total of 10 different areas per experiment were analyzed for the size of lipid droplets. Each measured area was comparable for all samples. The size of lipid droplets was measured using the Image J software, according to the reported methods [78]. 

### 4.6. Metabolomic Analysis

Inguinal ADSCs- and epididymal ADSCs-derived adipocytes, which were prepared by the above methods, were treated with isoproterenol (50 μM) for 120 min in the fresh DMEM. After the stimulation, the adipocytes were used for metabolomic analysis (Human Metabolome Technologies Inc., Tsuruoka, Japan). The adipocytes (10^6^) were transferred into 500 μL of methanol containing 50 mM external standard. After homogenization by BMSM10N21 (BMS, Tokyo) (2038 g for 120 s) five times, 500 μL of chloroform and 200 μL of ultrapure water were added to the homogenate, according to the protocol presented by Human Metabolome Technologies. The solution was mixed well and centrifuged at 2300× *g* for 5 min at 4 °C. The resultant water phase was ultrafiltered using a Millipore Ultrafree-MC PLHCC HMT Centrifugal Filter Device, 5 kDa (Millipore, Billerica, MA, USA). The filtrate was dried and dissolved in 50 μL of ultrapure water. The samples obtained were then subjected to CE-TOFMS analysis using the Agilent CE-TOFMS system (Agilent Technologies, Santa Clara, CA, USA) at 4 °C. The detected peaks were aligned according to their m/z values and normalized migration times. The peaks were mean-centered and scaled using standard deviations on a per peak basis as a pretreatment. After autoscaling, PCA and hierarchical clustering analysis (HCA) were conducted using SAMPLESTAT ver. 3.14 and PeakStat ver. 3.18 (Human Metabolome Technologies Inc.). In the PCA, a score plot of the first and second principal components was generated. In the HCA, the resulting datasets from each group were clustered via Euclidean distance using the Ward method. Heat maps were generated by coloring the data across their value ranges. The relative area of each peak was calculated and compared between groups. Metabolomics data were submitted to Metabolomics Workbench (www.metabolomicsworkbench.org) and MetaboAnalyst (www.metaboanalyst.ca).

### 4.7. Statistical Analysis 

The results of the animal physical characteristics and the cell size were analyzed using one-way analysis of variance (ANOVA), and where the main effects were considered significant, the Bonferroni post hoc test for multiple comparisons was conducted. For metabolome analysis, values were presented as the mean ± standard deviation (S.D.), and Welch’s *t*-test was performed to test the statistical significance between groups; the significance level was at *p* < 0.05, or less. The analyses were performed using Microsoft Excel (Redmond, WA, USA). 

## 5. Conclusions

The present data highlight that the preconditioning of either TR or HFD intake significantly affected the accumulation of metabolites in adipocytes differentiated from ADSCs in vitro. Changes in the metabolic profile of adipocytes due to TR and HFD are particularly noticeable via the accumulation of amino acids in an adipose region-specific fashion. Our observed modulation of cell metabolism of ADSCs could be associated with the reported changes in energy metabolism in the perigonadal and inguinal SVF-derived cells, as well as higher retention, higher proangiogenic milieu, and change in the differential behavior of ADSCs. The findings may provide a new insight into strategies for the treatment of obesity by exercise. Further knowledge of the underlying mechanisms is required to enhance superiority of exercise in the prevention and treatment of obesity.

## Figures and Tables

**Table 1:** The ratio of metabolites.

	Epididymal ADSC-Derived Adipocytes			Inguinal ADSC-Derived Adipocytes		
	ND-TR *vs*. ND-SED	HFD-SED *vs*. ND-SED	HFD-TR *vs*. HFD-SED	ND-TR *vs*. ND-SED	HFD-SED *vs*. ND-SED	HFD-TR *vs*. HFD-SED
GSH/GSSG	1.2	0.8	1.1	0.6 *	0.9	1.2 **
NADP^+^/NADPH	0.7	0.5	1.4	0.7	1.4	0.7
NAD^+^/NADH	0.8 *	0.7 *	0.9	0.7 *	1.4 *	0.7 *
Lactate/Pyruvate	1.1	1.4 *	1.0	0.8	0.8	1.0
Malate/Asp	1.1	1.3	1.0	0.6 ***	0.7 **	1.1
Citrulline/Ornithine	1.1	1.0	1.0	1.1	0.8	1.1
Glu/2-Oxoglutarate	1.3 **	1.5	1.5	1.7 *	1.1	1.5

* *p* < 0.05, ** *p* < 0.01, *** *p* < 0.001 vs. each respective control. GSH, glutathione, GSSG, glutathione disulfide; ND-SED, normal diet-fed sedentary controls; HFD-SED, high-fat diet-fed sedentary controls; ND-TR, normal diet-fed exercise-trained; HFD-TR, high-fat diet-fed exercise-trained group.

## Data Availability

The data that support the findings of this study are available from the corresponding author upon reasonable request.

## Supplementary Materials

The following are available online at https://www.mdpi.com/1422-0067/22/2/966/s1 Figure S1. The expression levels of ADSCs markers. Table S1. Relative ratio of metabolites in epididymal ADSC-derived adipocytes. Table S2. Relative ratio of metabolites in inguinal ADSCs-derived adipocytes.

## Abbreviations

ADSCs	adipose-derived stem/progenitor cells
BCAA	branched-chain amino acid
BODIPY	boron dipyrrin
DMEM	Dulbecco’s modified Eagle’s medium
ES	embryonic stem
HFD	high-fat diet
KEGG	Kyoto Encyclopedia of Genes and Genomes
ND	normal diet
PCA	principal component analysis
PGC1-α	peroxisome proliferator-activated receptor γ coactivator 1-α
PPAR	peroxisome proliferator-activated receptor
PRPP	5-Phosphoribosyl 1-diphosphate
SAT	subcutaneous white adipose tissue
SED	sedentary control
SVF	stromal vascular fraction
TR	exercise training
mTORC1	mammalian target of rapamycin complex 1
VAT	visceral adipose tissue
