# Supplementary material for: Metabolomic Profiles in Adipocytes Differentiated from Adipose-Derived Stem Cells Following Exercise Training or High-Fat Diet
Source: Int J Mol Sci. 2021 Jan 19;22(2):966. doi: 10.3390/ijms22020966 (PMC7835847; doi:10.3390/ijms22020966)
Supplement: Supplementary file 1 [file ijms-22-00966-s001.pdf]

（A）Epididymal ADSCs

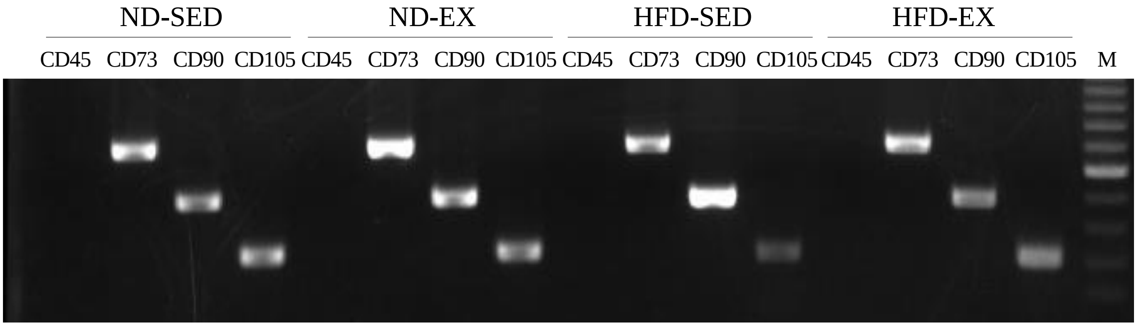

（B）Inguinal ADSCs

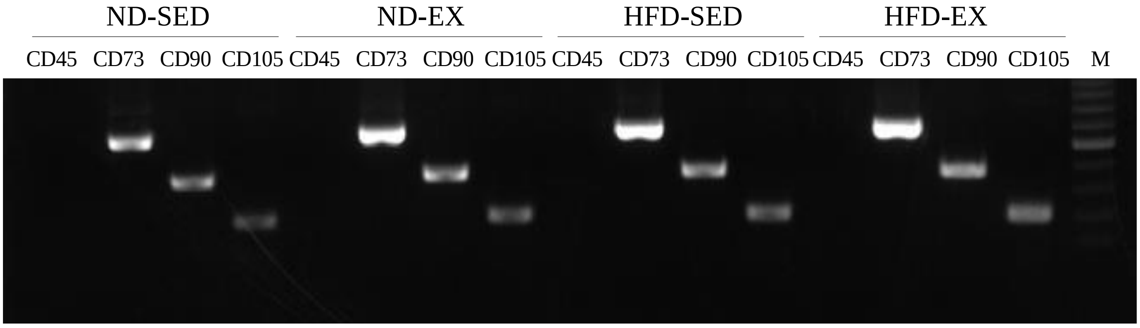

Figure S1. The expression levels of ADSCs markers. Reverse transcription-polymerase chain reaction (RT-PCR) was performed by using RNA extracted from ADSCs. CD45 used as a negative marker. CD73, CD90, and CD105 were used as positive markers for the analysis of ADSCs. **A**. Epididymal ADSCs. **B**. Inguinal ADSCs.

Table S1. Relative ratio of metabolites in epididymal ADSC-derived adipocytes.

Compound name	Comparative Analysis								
	ADSC-epi-ND-TR vs ADSC-epi-ND-SED			ADSC-epi-HFD-SED vs ADSC-epi-ND-SED			ADSC-epi-HFD-TR vs ADSC-epi-HFD-SED		
	Ratio ¶	*p*-value ‖		Ratio ¶	*p*-value ‖		Ratio ¶	*p*-value ‖	
NAD+	1.1	0.51		1.1	0.53		1.2	0.15	
cAMP	1.1	0.23		1.1	0.72		1.4	0.29	
cGMP	N.A.	N.A.		N.A.	N.A.		N.A.	N.A.	
NADH	1.4	0.17		1.5	0.11		1.3	0.11	
Xanthine	1.1	0.26		0.8	0.25		1.4	0.44	
ADP-ribose	1.1	0.58		1.3	0.09		0.8	0.23	
Mevalonic acid	N.A.	N.A.		N.A.	N.A.		N.A.	N.A.	
UDP-glucose	1.1	0.65		1.1	0.67		1.1	0.27	
Uric acid	1.1	0.47		1.0	0.71		1.2	0.34	
NADP+	1.1	0.59		0.9	0.62		1.4	0.20	
IMP	1.3	0.39		1.0	0.98		2.1	0.15	
Sedoheptulose 7-phosphate	N.A.	N.A.		1<	N.A.		27	N.A.	
Glucose 6-phosphate	1.2	0.04	*	1.2	0.15		1.1	0.70	
Fructose 6-phosphate	1.5	0.04	*	1.4	0.20		1.8	0.20	
Fructose 1-phosphate	1.0	0.73		1.4	0.05	*	1.2	0.17	
Galactose 1-phosphate	0.8	0.29		1.1	0.57		1.0	0.81	
Glucose 1-phosphate	0.9	0.66		1.3	0.23		0.9	0.63	
Acetoacetyl CoA	N.A.	N.A.		N.A.	N.A.		N.A.	N.A.	
Acetyl CoA	1.2	0.63		1.2	0.61		2.7	0.40	
Folic acid	1.2	0.37		1.6	0.08		1.0	0.84	
Ribose 5-phosphate	1.2	0.66		2.7	0.12		1.6	0.23	
CoA	1.1	0.55		1.2	0.58		1.1	0.74	
Ribose 1-phosphate	1.7	0.30		1.1	0.90		1.6	0.67	
Ribulose 5-phosphate	1.3	0.44		2.7	0.05	*	1.5	0.15	
Xylulose 5-phosphate	3.3	0.11		13	0.05	*	1.5	0.37	
Erythrose 4-phosphate	1<	N.A.		1<	N.A.		1.9	N.A.	
HMG CoA	1.0	0.76		1.5	0.03	*	1.1	0.27	
Glyceraldehyde 3-phosphate	1.6	0.32		2.4	0.07		2.0	0.19	
NADPH	1.5	0.29		1.4	0.50		1.2	0.66	
Malonyl CoA	1.3	0.50		1.1	0.84		1.9	0.18	
Phosphocreatine	1.1	0.30		1.2	0.12		1.3	0.23	
XMP	1.2	0.58		1.4	0.19		1.7	0.25	
Dihydroxyacetone phosphate	1.3	0.39		2.0	0.10		1.6	0.24	
Adenylosuccinic acid	1.2	0.30		1.2	0.30		2.1	0.16	
Fructose 1,6-diphosphate	1.5	0.15		2.2	0.06		1.8	0.13	
6-Phosphogluconic acid	1.2	0.08		1.5	0.01	*	1.2	0.12	
N-Carbamoylaspartic acid	1.8	0.08		2.2	0.06		1.3	0.18	
PRPP	0.8	0.21		0.6	0.01	**	1.1	0.83	
2-Phosphoglyceric acid	1.1	0.21		1.2	0.06		1.1	0.13	
2,3-Diphosphoglyceric acid	1.8	0.00	**	1.9	0.02	*	1.3	0.30	
3-Phosphoglyceric acid	1.0	0.46		1.1	0.10		1.2	0.15	
Phosphoenolpyruvic acid	1.2	0.17		1.0	0.89		1.4	0.05	*
GMP	1.3	0.04	*	1.0	0.88		1.5	0.08	
AMP	1.2	0.24		1.2	0.12		1.4	0.28	
2-Oxoisovaleric acid	1.1	0.49		1.1	0.27		1.2	0.34	
GDP	1.5	0.01	*	1.1	0.19		1.5	0.01	**

Lactic acid	1.3	0.03	*	1.2	0.09		1.3	0.04	*
ADP	1.3	0.02	*	1.0	0.83		1.6	0.01	**
GTP	1.3	0.07		1.3	0.05	*	1.2	0.03	*
Glyoxylate	N.A.	N.A.		N.A.	N.A.		N.A.	N.A.	
ATP	1.2	0.05	*	1.1	0.15		1.2	0.06	
Glycerol 3-phosphate	1.3	0.09		1.9	0.00	**	1.0	0.80	
Glycolic acid	1.4	N.A.		1.0	N.A.		0.14	N.A.	
Pyruvic acid	1.1	0.08		0.9	0.10		1.2	0.12	
N-Acetylglutamic acid	1.0	0.78		1.1	0.22		1.1	0.41	
2-Hydroxyglutaric acid	1.2	0.17		1.3	0.20		1.1	0.65	
Carbamoylphosphate	1<	N.A.		N.A.	N.A.		N.A.	N.A.	
Succinic acid	1.3	0.30		0.9	0.74		1.2	0.35	
Malic acid	1.4	0.04	*	1.3	0.01	*	1.2	0.03	*
2-Oxoglutaric acid	1.1	0.47		0.8	0.08		1.0	1.00	
Fumaric acid	1.5	0.01	**	1.4	0.06		1.2	0.14	
Citric acid	1.1	0.11		0.8	0.05	*	1.1	0.22	
cis-Aconitic acid	1.2	0.20		0.8	0.08		1.1	0.71	
Isocitric acid	2.0	0.17		0.3	N.A.		2.3	N.A.	
Urea	N.A.	N.A.		N.A.	N.A.		N.A.	N.A.	
Gly	1.2	0.02	*	1.0	0.40		1.2	0.04	*
Putrescine	0.9	0.65		0.6	0.12		<1	N.A.	
Ala	1.4	0.01	**	1.0	0.90		1.3	0.00	**
β-Ala	1.2	0.16		0.8	0.04	*	1.3	0.07	
Sarcosine	N.A.	N.A.		N.A.	N.A.		N.A.	N.A.	
N,*N*-Dimethylglycine	N.A.	N.A.		N.A.	N.A.		N.A.	N.A.	
γ-Aminobutyric acid	1.2	0.13		1.0	0.52		1.1	0.04	*
Choline	1.7	0.03	*	1.9	0.02	*	1.2	0.26	
Ser	1.2	0.04	*	0.8	0.02	*	1.3	0.00	**
Carnosine	1.4	0.21		1.0	0.92		1.3	0.18	
Creatinine	1.1	0.76		1.2	0.43		0.9	0.70	
Pro	1.3	0.63		0.6	0.58		2.3	0.13	
Val	1.4	0.02	*	1.1	0.17		1.3	0.00	**
Betaine	1.2	0.12		1.4	0.03	*	0.9	0.32	
Homoserine	1<	N.A.		N.A.	N.A.		N.A.	N.A.	
Thr	1.2	0.02	*	0.8	0.02	*	1.3	0.02	*
Betaine aldehyde	N.A.	N.A.		N.A.	N.A.		N.A.	N.A.	
Cys	N.A.	N.A.		N.A.	N.A.		N.A.	N.A.	
Hydroxyproline	1.2	0.15		0.8	0.15		1.2	0.00	***
Creatine	1.2	0.12		1.3	0.20		1.3	0.31	
Ile	1.5	0.01	*	1.2	0.12		1.4	0.02	*
Leu	1.4	0.05	*	1.2	0.14		1.3	0.04	*
Asn	1.2	0.30		0.9	0.33		1.2	0.08	
Ornithine	1.1	0.73		0.8	0.18		1.2	0.05	*
Asp	1.3	0.02	*	1.0	0.56		1.3	0.01	*
Homocysteine	N.A.	N.A.		N.A.	N.A.		N.A.	N.A.	
Adenine	1.2	0.35		1.1	0.60		1.9	0.00	**
Hypoxanthine	1.1	0.26		0.7	0.05	*	1.4	0.07	
Spermidine	N.A.	N.A.		N.A.	N.A.		N.A.	N.A.	
Gln	1.2	0.01	**	0.8	0.00	***	1.2	0.02	*
Lys	1.1	0.02	*	1.0	0.20		1.1	0.19	
Glu	1.3	0.01	*	1.2	0.11		1.1	0.08	
Met	1.4	0.05	*	1.0	0.71		1.4	0.01	*
Guanine	N.A.	N.A.		N.A.	N.A.		N.A.	N.A.	
His	1.2	0.03	*	1.0	0.98		1.1	0.06	

Carnitine	0.8	0.76		2.6	0.18		1.1	N.A.	
Phe	1.5	0.02	*	1.1	0.29		1.4	0.01	**
Arg	1.1	0.17		0.9	0.33		1.1	0.18	
Citrulline	1.2	0.02	*	0.8	0.02	*	1.2	0.03	*
Tyr	1.3	0.01	**	1.0	0.39		1.3	0.01	**
S-Adenosylhomocysteine	0.8	N.A.		0.5	N.A.		1.1	N.A.	
Spermine	0.7	0.05	*	3.6	0.03	*	0.9	0.65	
Trp	1.4	0.02	*	1.1	0.22		1.3	0.01	*
Cystathionine	1.3	0.01	**	1.1	0.03	*	1.2	0.17	
Adenosine	1.1	0.53		0.9	0.31		1.3	0.39	
Inosine	1.2	0.27		1.1	0.50		1.5	0.40	
Guanosine	1.2	0.37		1.0	0.94		1.3	0.42	
Argininosuccinic acid	1.2	0.09		0.9	0.32		1.1	0.15	
Glutathione (GSSG)	1.1	0.44		1.1	0.54		1.1	0.66	
Glutathione (GSH)	1.4	0.16		0.9	0.65		1.2	0.17	
S-Adenosylmethionine	1.3	0.01	**	1.0	0.57		1.2	0.03	*
Adenylate Energy Charge	1.0	0.74		1.0	0.40		1.0	0.17	
Total Adenylate	1.2	0.04	*	1.1	0.16		1.2	0.04	*
Guanylate Energy Charge	1.0	0.40		1.0	0.16		1.0	0.15	
Total Guanylate	1.3	0.05		1.3	0.05		1.2	0.01	*
GSH/GSSG	1.2	0.55		0.8	0.59		1.1	0.70	
Total Glutathione	1.3	0.09		1.0	0.81		1.2	0.04	*
NADPH/NADP+	1.5	0.18		2.2	0.41		0.7	0.59	
NADH/NAD+	1.3	0.05	*	1.4	0.03	*	1.1	0.23	
Lactate/Pyruvate	1.1	0.27		1.4	0.04	*	1.0	0.66	
Glycerol 3-phosphate/DHAP	0.9	0.82		0.9	0.77		0.7	0.18	
Total Amino Acids	1.2	0.01	**	0.9	0.17		1.2	0.00	***
Total Essential Amino Acids	1.3	0.01	*	0.9	0.11		1.3	0.00	**
Total Non-essential Amino Acids	1.2	0.01	**	0.9	0.19		1.2	0.00	***
Total Glucogenic Amino Acids	1.2	0.01	**	0.9	0.16		1.2	0.00	***
Total Ketogenic Amino Acids	1.2	0.01	*	0.9	0.07		1.3	0.00	**
Total BCAA	1.4	0.03	*	1.2	0.12		1.3	0.01	**
Total Aromatic Amino Acids	1.4	0.01	*	1.1	0.31		1.3	0.01	**
Fischer's Ratio	1.1	0.15		1.1	0.04	*	1.0	0.29	
Total Glu-related Amino Acids	1.2	0.01	**	0.9	0.24		1.2	0.00	***
Total Pyr-related Amino Acids	1.2	0.01	*	0.9	0.08		1.3	0.00	**
Total Acetyl CoA-related Amino Acids	1.3	0.02	*	1.1	0.24		1.2	0.02	*
Total Fumarate-related Amino Acids	1.4	0.01	*	1.1	0.33		1.3	0.01	**
Total Succinyl CoA-related Amino Acids	1.4	0.02	*	1.1	0.15		1.3	0.01	**
Total Oxaloacetate-related Amino Acids	1.3	0.02	*	1.0	0.59		1.3	0.01	*
Malate/Asp	1.1	0.59		1.3	0.10		1.0	0.57	
Citrulline/Ornithine	1.1	0.50		1.0	0.98		1.0	0.78	
Glu/2-Oxoglutarate	1.3	0.01	**	1.5	0.06		1.2	0.53	
G6P/R5P	0.8	0.69		0.4	0.30		0.6	0.44	
SAM/SAH	1.6	N.A.		2.1	N.A.		1.1	N.A.	
Putrescine/Spermidine	N.A.	N.A.		N.A.	N.A.		N.A.	N.A.	

N.A.: Not available. Although it was a target for calculation, it could not be calculated due to lack of data. The ratio of the detected mean values between the two groups was calculated with the latter as the denominator. Welch's t-test *p*-value and its range are shown. (**p* < 0.05, **p < 0.01, *** *p* < 0.001)

Table S2. Relative ratio of metabolites in inguinal ADSC-derived adipocytes.

HMT DB	Comparative Analysis								
Compound name	ADSC-ing-ND-TR vs ADSC-ing-ND-SED			ADSC-ing-HFD-SED vs ADSC-ing-ND-SED			ADSC-ing-HFD-TR vs ADSC-ing-HFD-SED		
	Ratio [¶]	*p*-value [‖]		Ratio [¶]	*p*-value [‖]		Ratio [¶]	*p*-value [‖]	
NAD^+	0.7	0.08		1.3	0.23		0.9	0.52	
cAMP	0.9	0.11		0.9	0.74		0.8	0.39	
cGMP	N.A.	N.A.		N.A.	N.A.		N.A.	N.A.	
NADH	1.0	0.82		0.9	0.44		1.3	0.08	
Xanthine	1.2	0.43		1.6	0.16		0.8	0.30	
ADP-ribose	0.7	0.08		1.0	0.88		1.0	0.93	
Mevalonic acid	N.A.	N.A.		N.A.	N.A.		N.A.	N.A.	
UDP-glucose	1.0	0.67		1.1	0.44		1.0	0.96	
Uric acid	1.2	0.21		1.4	0.17		1.2	0.47	
$NADP^+$	0.7	0.08		1.2	0.17		1.1	0.70	
IMP	1.0	0.97		3.1	0.02	*	1.0	0.83	
Sedoheptulose 7-phosphate	N.A.	N.A.		N.A.	N.A.		N.A.	N.A.	
Glucose 6-phosphate	1.1	0.37		1.3	0.00	**	1.2	0.05	*
Fructose 6-phosphate	1.8	0.03	*	1.8	0.02	*	1.6	0.11	
Fructose 1-phosphate	1.6	0.12		1.8	0.07		1.6	0.11	
Galactose 1-phosphate	0.4	0.01	**	1.1	0.74		0.7	0.29	
Glucose 1-phosphate	0.4	0.00	***	1.0	0.85		0.8	0.24	
Acetoacetyl CoA	N.A.	N.A.		N.A.	N.A.		N.A.	N.A.	
Acetyl CoA	0.8	0.07		0.8	0.43		2.4	0.16	
Folic acid	1.4	0.08		1.2	0.35		1.1	0.44	
Ribose 5-phosphate	1.6	0.09		1.7	0.22		1.5	0.23	
CoA	0.4	0.05	*	1.0	0.90		0.9	0.34	
Ribose 1-phosphate	1<	N.A.		1<	N.A.		5.2	0.14	
Ribulose 5-phosphate	1.7	0.06		1.5	0.02	*	1.4	0.17	
Xylulose 5-phosphate	2.3	0.12		2.0	0.17		2.0	0.23	
Erythrose 4-phosphate	<1	N.A.		3.3	N.A.		0.9	N.A.	
HMG CoA	0.9	0.51		0.8	0.09		1.2	0.14	
Glyceraldehyde 3-phosphate	1.8	0.14		2.1	0.04	*	2.1	0.04	*
NADPH	1.0	0.90		0.9	0.73		1.6	0.12	
Malonyl CoA	N.A.	N.A.		N.A.	N.A.		1<	N.A.	
Phosphocreatine	0.7	0.06		1.2	0.29		1.1	0.53	
XMP	1.9	0.04	*	3.2	0.03	*	1.4	0.14	
Dihydroxyacetone phosphate	1.7	0.00	***	2.0	0.02	*	1.6	0.14	
Adenylosuccinic acid	0.8	0.34		1.6	0.08		1.0	0.85	
Fructose 1,6-diphosphate	2.3	0.01	**	2.7	0.02	*	1.6	0.06	
6-Phosphogluconic acid	0.9	0.21		1.2	0.36		1.0	0.91	
N-Carbamoylaspartic acid	0.6	0.06		0.7	0.07		0.8	0.34	
PRPP	0.9	0.48		2.1	0.02	*	0.7	0.11	
2-Phosphoglyceric acid	1.0	0.78		1.0	0.68		1.1	0.63	
2,3-Diphosphoglyceric acid	7.0	0.01	*	0.8	0.27		2.9	0.00	**
3-Phosphoglyceric acid	1.0	0.87		1.1	0.51		1.2	0.20	
Phosphoenolpyruvic acid	1.1	0.55		1.2	0.20		1.3	0.21	
GMP	0.6	0.05	*	1.1	0.58		1.1	0.29	
AMP	0.5	0.00	***	1.2	0.03	*	1.0	0.90	
2-Oxoisovaleric acid	1.0	0.69		1.5	0.00	**	0.8	0.01	*

GDP	0.7	0.11		1.2	0.34		0.9	0.17	
Lactic acid	1.0	0.92		1.2	0.17		0.9	0.42	
ADP	0.7	0.11		1.2	0.30		0.9	0.25	
GTP	0.8	0.10		1.2	0.33		1.0	0.76	
Glyoxylate	N.A.	N.A.		N.A.	N.A.		N.A.	N.A.	
ATP	0.8	0.06		1.1	0.31		1.0	0.81	
Glycerol 3-phosphate	0.9	0.25		1.1	0.45		0.9	0.50	
Glycolic acid	1.7	0.36		1.7	0.31		<1	N.A.	
Pyruvic acid	1.2	0.05	*	1.4	0.04	*	1.0	0.60	
N-Acetylglutamic acid	0.9	0.28		1.1	0.42		0.9	0.50	
2-Hydroxyglutaric acid	0.6	0.00	***	1.0	0.76		0.8	0.22	
Carbamoylphosphate	N.A.	N.A.		N.A.	N.A.		N.A.	N.A.	
Succinic acid	0.7	0.06		1.3	0.01	**	1.1	0.14	
Malic acid	0.5	0.00	**	0.9	0.13		1.0	0.98	
2-Oxoglutaric acid	0.6	0.01	*	1.0	0.93		0.7	0.02	*
Fumaric acid	0.4	0.00	**	0.9	0.13		0.9	0.12	
Citric acid	0.7	0.04	*	1.1	0.34		1.0	0.90	
cis-Aconitic acid	0.5	0.05	*	1.0	0.46		0.9	0.15	
Isocitric acid	0.2	0.24		1.2	0.72		0.7	0.50	
Urea	<1	N.A.		1.3	0.11		0.9	0.09	
Gly	0.7	0.00	**	1.3	0.01	**	1.0	0.77	
Putrescine	1.4	0.25		1.0	1.00		1.0	0.88	
Ala	0.7	0.04	*	1.5	0.01	**	1.0	0.54	
β-Ala	0.7	0.03	*	1.2	0.06		1.0	0.81	
Sarcosine	N.A.	N.A.		N.A.	N.A.		N.A.	N.A.	
γ-Aminobutyric acid	1.2	0.26		1.0	0.99		1.1	0.11	
N,*N*-Dimethylglycine	N.A.	N.A.		N.A.	N.A.		N.A.	N.A.	
Choline	1.0	0.99		1.5	0.00	***	1.2	0.09	
Ser	0.7	0.01	*	1.1	0.06		1.0	0.21	
Carnosine	0.6	0.08		1.4	0.07		0.9	0.26	
Creatinine	0.8	0.12		1.4	0.05	*	0.9	0.36	
Pro	0.7	0.01	*	1.5	0.01	**	1.0	0.68	
Val	0.8	0.02	*	1.4	0.00	**	1.0	0.91	
Betaine	<1	N.A.		1.5	0.25		1.1	0.85	
Thr	0.7	0.01	*	1.3	0.01	**	0.9	0.10	
Homoserine	N.A.	N.A.		N.A.	N.A.		1<	N.A.	
Betaine aldehyde	N.A.	N.A.		N.A.	N.A.		N.A.	N.A.	
Cys	N.A.	N.A.		1<	N.A.		2.0	N.A.	
Hydroxyproline	0.7	0.08		1.5	0.03	*	0.9	0.01	*
Creatine	0.6	0.07		1.7	0.02	*	0.8	0.02	*
Ile	0.8	0.03	*	1.4	0.01	*	1.0	0.51	
Leu	0.8	0.03	*	1.4	0.00	**	1.0	0.86	
Asn	0.5	0.01	**	1.3	0.04	*	0.9	0.06	
Ornithine	0.8	0.13		1.7	0.00	**	0.9	0.01	*
Asp	0.9	0.22		1.3	0.00	***	0.9	0.51	
Homocysteine	N.A.	N.A.		N.A.	N.A.		N.A.	N.A.	
Adenine	<1	N.A.		<1	N.A.		N.A.	N.A.	
Hypoxanthine	<1	N.A.		1.6	0.03	*	0.8	N.A.	
Spermidine	0.02	N.A.		1.0	N.A.		1.6	0.35	
Gln	0.7	0.02	*	1.4	0.01	*	0.9	0.13	
Lys	0.7	0.02	*	1.7	0.00	**	0.9	0.12	
Glu	1.1	0.48		1.1	0.01	*	1.0	0.65	
Met	0.8	0.01	**	1.4	0.00	***	1.0	0.47	
Guanine	N.A.	N.A.		N.A.	N.A.		N.A.	N.A.	

His	0.7	0.01	*	1.3	0.01	**	1.0	0.44	
Carnitine	N.A.	N.A.		N.A.	N.A.		N.A.	N.A.	
Phe	0.7	0.04	*	1.4	0.02	*	0.9	0.21	
Arg	0.7	0.02	*	1.6	0.00	**	0.9	0.17	
Citrulline	0.9	0.40		1.4	0.00	**	1.0	0.59	
Tyr	0.7	0.03	*	1.4	0.02	*	0.9	0.18	
S-Adenosylhomocysteine	0.9	N.A.		1.0	0.74		1.1	0.72	
Spermine	N.A.	N.A.		N.A.	N.A.		N.A.	N.A.	
Trp	0.7	0.04	*	1.5	0.01	*	0.9	0.08	
Cystathionine	0.7	0.27		0.7	0.03	*	0.9	0.13	
Adenosine	0.7	0.01	**	1.2	0.03	*	1.0	0.89	
Inosine	0.9	0.43		2.7	0.01	**	1.0	0.94	
Guanosine	0.8	0.02	*	1.6	0.03	*	1.0	0.80	
Argininosuccinic acid	0.5	0.02	*	1.4	0.03	*	0.8	0.07	
Glutathione (GSSG)	1.0	0.87		1.4	0.00	***	0.9	0.01	**
Glutathione (GSH)	0.6	0.01	*	1.2	0.05	*	1.0	0.37	
S-Adenosylmethionine	0.8	0.10		1.1	0.25		1.0	0.80	
Adenylate Energy Charge	1.0	0.04	*	1.0	0.20		1.0	0.19	
Total Adenylate	0.7	0.05		1.1	0.29		1.0	0.74	
Guanylate Energy Charge	1.0	0.14		1.0	0.72		1.0	0.71	
Total Guanylate	0.8	0.09		1.1	0.32		1.0	0.74	
GSH/GSSG	0.6	0.03	*	0.9	0.36		1.2	0.00	**
Total Glutathione	0.8	0.01	**	1.3	0.00	**	1.0	0.43	
NADPH/NADP+	1.5	0.10		0.7	0.32		1.5	0.18	
NADH/NAD+	1.5	0.01	*	0.7	0.03	*	1.5	0.01	*
Lactate/Pyruvate	0.8	0.11		0.8	0.11		1.0	0.57	
Glycerol 3-phosphate/DHAP	0.5	0.01	**	0.6	0.01	*	0.6	0.07	
Total Amino Acids	0.8	0.01	*	1.3	0.01	**	0.9	0.12	
Total Essential Amino Acids	0.7	0.02	*	1.4	0.01	*	0.9	0.03	*
Total Non-essential Amino Acids	0.8	0.01	*	1.3	0.01	**	1.0	0.16	
Total Glucogenic Amino Acids	0.8	0.01	*	1.3	0.01	**	1.0	0.12	
Total Ketogenic Amino Acids	0.7	0.02	*	1.4	0.01	*	0.9	0.02	*
Total BCAA	0.8	0.02	*	1.4	0.01	**	1.0	0.83	
Total Aromatic Amino Acids	0.7	0.04	*	1.5	0.02	*	0.9	0.16	
Fischer's Ratio	1.2	0.01	*	1.0	0.34		1.1	0.04	*
Total Glu-related Amino Acids	0.8	0.03	*	1.3	0.01	*	0.9	0.11	
Total Pyr-related Amino Acids	0.7	0.01	**	1.3	0.01	**	1.0	0.15	
Total Acetyl CoA-related Amino Acids	0.8	0.03	*	1.5	0.01	**	1.0	0.43	
Total Fumarate-related Amino Acids	0.7	0.03	*	1.4	0.02	*	0.9	0.17	
Total Succinyl CoA-related Amino Acids	0.8	0.02	*	1.4	0.01	**	1.0	0.80	
Total Oxaloacetate-related Amino Acids	0.8	0.18		1.3	0.00	***	0.9	0.48	
Malate/Asp	0.6	0.00	***	0.7	0.00	**	1.1	0.58	
Citrulline/Ornithine	1.1	0.28		0.8	0.06		1.1	0.27	
Glu/2-Oxoglutarate	1.7	0.02	*	1.1	0.17		1.5	0.08	
G6P/R5P	0.6	0.21		0.8	0.50		0.8	0.48	
SAM/SAH	0.9	N.A.		1.1	0.21		1.0	0.88	
Putrescine/Spermidine	90	N.A.		28	N.A.		0.03	0.42	

N.A.: Not available. Although it was a target for calculation, it could not be calculated due to lack of data. The ratio of the detected mean values between the two groups was calculated with the latter as the denominator. Welch's t-test *p*-value and its range are shown. (**p* <0.05, ***p* <0.01, ****p* <0.001)
